# Early Signs of Microvascular Endothelial Dysfunction in Adolescents with Newly Diagnosed Essential Hypertension

**DOI:** 10.3390/life12071048

**Published:** 2022-07-13

**Authors:** Tomas Jurko, Michal Mestanik, Andrea Mestanikova, Kamil Zeleňák, Alexander Jurko

**Affiliations:** 1 Clinic of Neonatology, Jessenius Faculty of Medicine in Martin (JFM CU), Comenius University in Bratislava, University Hospital Martin, Kollarova 2, 03659 Martin, Slovakia; jurko3@uniba.sk; 2 Pediatric Cardiology Clinic, Kollarova 13, 03601 Martin, Slovakia; mestanik@gmail.com (M.M.); andrea.mestanikova@gmail.com (A.M.); 3 Clinic of Radiology, Jessenius Faculty of Medicine in Martin, Comenius University in Bratislava, Kollarova 2, 03659 Martin, Slovakia; kamil.zelenak@uniba.sk

**Keywords:** endothelial function, blood pressure, essential hypertension, white-coat hypertension, adolescence, peripheral arterial tonometry

## Abstract

Endothelial dysfunction represents one of the key pathomechanisms in many diseases, including hypertension. Peripheral arterial tonometry (PAT) evaluates the functional status of microvascular endothelium and offers a biomarker of early, potentially reversible, vascular damage. This study aimed to assess endothelial function using conventional and novel indices of PAT in pediatric hypertensives. As such, 100 adolescents with normal blood pressure, and essential and white-coat hypertension were examined using EndoPAT 2000. Conventional reactive hyperemia index (RHI) and novel indices of hyperemic response, including the area under the curve of hyperemic response (AUC), were evaluated. AUC was the only parameter sensitive to the effect of hypertension, with significantly lower values in essential hypertensives compared to normotensives and white-coat hypertensives (*p* = 0.024, *p* = 0.032, respectively). AUC was the only parameter significantly correlating with mean ambulatory monitored blood pressure (*r* = −0.231, *p* = 0.021). AUC showed a significant negative association with age (*p* = 0.039), but a significant positive association with pubertal status indexed by plasma levels of dehydroepiandrosterone (*p* = 0.027). This is the first study reporting early signs of microvascular endothelial dysfunction evaluated using PAT in adolescents with newly diagnosed essential hypertension. Detailed analysis of hyperemic response using overall magnitude indexed by AUC provided a more robust method compared to the conventional evaluation of RHI.

## 1. Introduction

In recent decades, endothelial dysfunction has been recognized as one of the key pathogenic mechanisms in many acute and chronic cardiovascular, pulmonary, metabolic, oncological, and infectious diseases [1]. Impaired function of the endothelium is characterized by altered regulation of vascular tone, cellular adhesion, coagulation, smooth muscle cell proliferation, and vessel wall inflammation, with nitric oxide (NO) representing the central mediator in this pathway [1,2]. Decreased bioavailability of NO reflects the early stage of vascular disease preceding the morphological changes and offers a biomarker of potentially reversible damage, which can be targeted to improve future health outcomes [3,4].

Endothelial function can be assessed using various methods, which differ in the evaluated vascular bed, physiological signals, invasiveness, operator dependency, and degree of other physiological mechanisms evaluated in addition to NO [5,6]. Peripheral arterial tonometry (PAT) uses the mechanism of reactive hyperemia to evaluate the functional status of the microvascular endothelium and it was suggested to present a more complex and earlier marker of dysfunction compared to macrovascular methods (e.g., flow-mediated dilation, FMD, evaluated using ultrasonography) [7,8]. Moreover, it offers an operator-independent method that is convenient and easy to use. The outcome of PAT is reactive hyperemia index (RHI), which is calculated as a change in pulse wave amplitude (PWA) during a fixed-time interval after blood-flow occlusion compared to baseline [9]. In children and adolescents, the endothelium is still under development, which may result in considerable variability in the timing of the hyperemic response. Therefore, evaluation of the overall curve of hyperemic response was suggested to better reflect the true endothelial function [10].

It is well known that the process of endothelial dysfunction begins early in life and is considered a marker of overall atherosclerotic risk resulting from the joint effect of single risk factors, e.g., hypertension, hyperlipidemia, diabetes mellitus, and others [4,7,11]. Several studies used PAT in the pediatric population and showed an increase in endothelial function with pubertal advancement and regular physical activity, and a decrease with pathological conditions, such as acute systemic vasculitis, sleep apnea syndrome, diabetes, insulin resistance, and obesity [12,13,14,15,16,17,18,19]. In hypertension, the connection is likely bidirectional—elevated blood pressure (BP) may both cause and be a consequence of endothelial dysfunction [20]. Regarding pediatric hypertension, altered macrovascular endothelial function evaluated using FMD was found already in adolescents with newly diagnosed essential and white-coat hypertension [21]. Yet, studies on microvascular endothelial function are still lacking. Therefore, we aimed to study endothelial function assessed using PAT in normal-weight and overweight adolescents with essential and white-coat hypertension, with a focus on conventional and novel indices of reactive hyperemia as potential early markers of vascular damage in the pediatric population.

## 2. Materials and Methods

### 2.1. Subjects

The study population consisted of 100 adolescents (79 boys) aged 12–19 years (16.3 ± 1.7 years, mean ± standard error) including 27 normotensives (24 normal-weight, 3 overweight, age 15.9 ± 1.9 years), 28 white-coat hypertensives (21 normal-weight, 7 overweight, age 16.7 ± 1.5 years), and 45 essential hypertensives (12 normal-weight, 33 overweight, age 16.3 ± 1.6 years). All probands were non-smokers with no history of recent acute illness or chronic cardiovascular, respiratory, endocrine (including diabetes mellitus), neurological, infectious diseases or mental disorders. They were instructed to avoid physical exercise and consumption of substances that could affect the cardiovascular or autonomic nervous system (e.g., medicaments, caffeine, and alcohol) 24 h prior to the examination. 

#### 2.1.1. Anthropometric Measures

Height (*h*, m) and weight (*w*, kg) were measured, and body mass index (BMI, kg/m^2^) was calculated as BMI = *w*/*h*^2^. The corresponding adult BMI was assessed for each participant aged <18 years as the BMI value potentially reached at age 18 years if the subject’s BMI would remain at the same percentile level according to the Extended International Obesity Task Force reference values [22]. 

#### 2.1.2. Diagnosis of Hypertension

Essential and white-coat hypertension was diagnosed by a specialist in pediatric cardiology according to the guidelines of the European Society of Hypertension for diagnosis and management of hypertension in childhood and adolescence [23]. Hypertension was defined as systolic BP and/or diastolic BP higher or equal to the 95th percentile adjusted for gender, age, and height [24]. BP was measured on three separate occasions using the auscultatory method. Afterward, 24 h ambulatory BP monitoring was applied to confirm/reject the diagnosis of essential hypertension and to identify the patients with white-coat hypertension defined by BP in the hypertensive range in the office but in the normotensive range (lower than the 90th percentile) outside the clinical settings [24]. Secondary hypertension was excluded in the specialized pediatric cardiology office for all the patients. As such, 24 h ambulatory BP monitoring was also applied in the normotensive group and mean BP from the whole 24 h was included in statistical analyses.

#### 2.1.3. Diagnosis of Overweight

Normal weight and overweight were defined according to the Extended International Obesity Task Force (IOTF) body mass index cut-offs for thinness, overweight, and obesity using age- and sex-specific body mass index (BMI) cut-offs, which correspond to the adult BMI range between 18.5 and 25 kg m^−2^ for normal weight and the threshold of 25 kg m^−2^ for overweight [22]. 

### 2.2. Protocol

The examinations were performed between 8.00 and 10.00 a.m. under standard conditions (temperature of the room 22 °C, minimalization of stimuli) and under fasting conditions. Blood samples were taken 30 min prior to the tests for serum glucose (mmol/L), lipids (high-density lipoprotein—HDL, low-density lipoprotein—LDL, triacylglycerols—TG; mmol/L), liver function tests—aspartate aminotransferase (AST, µkat/L), alanine aminotransferase (ALT, µkat/L), gamma-glutamyl transferase (GGT, µkat/L), alkaline phosphatase (ALP, µkat/L), and a laboratory indicator of pubertal development, dehydroepiandrosterone sulphate (DHEAS, µmol/L) [25]. The samples were analyzed in an external laboratory accredited in accordance with ISO 15 189:2007.

Before the examination, BP was measured and the participants were rested in supine position for 15 min. They were instructed not to speak or sleep during the examination. Afterward, microvascular endothelial function was assessed by the PAT method using the system EndoPAT 2000 (Itamar Medical, Caesarea, Israel) based on the plethysmographic assessment of PWA. Pneumatic probes (applying a counter pressure of 70 mmHg to avoid venous distention and related reflex responses [26]) were placed on the index fingers of both hands. Continuous recording consisted of three 5 min phases: baseline, ischemic phase, and postischemic phase. Reactive hyperemia was induced by inflation of the pressure cuff placed on the non-dominant arm before the beginning of the ischemic period. Inflation pressure was calculated as systolic BP + 60 mmHg, minimally 200 mmHg. After this period, the cuff was rapidly deflated and the postischemic signal was recorded.

### 2.3. Evaluated Parameters of Endothelial Function

*Reactive hyperemia index (RHI)* was calculated using the automated algorithm from the PWA recording as the post-to-pre occlusion signal ratio in the occluded arm, normalized to the control arm, and corrected for baseline vascular tone, using a fixed interval 90–150 s within post-occlusion period [9]. A natural log transformation of this parameter—*LnRHI*—is preferred since it provides double-sided distribution closer to normal distribution. 

*FRHI* is an index used in the Framingham Heart Studies. It is calculated as a natural log transform of the ratio between the post- to pre-occluded PAT amplitudes and the same ratio of the PAT amplitudes measured at the control arm. FRHI does not incorporate a correction to the baseline and uses shorter post occlusion times (1.5–2 min) than RHI [9,27].

**Figure 1:**
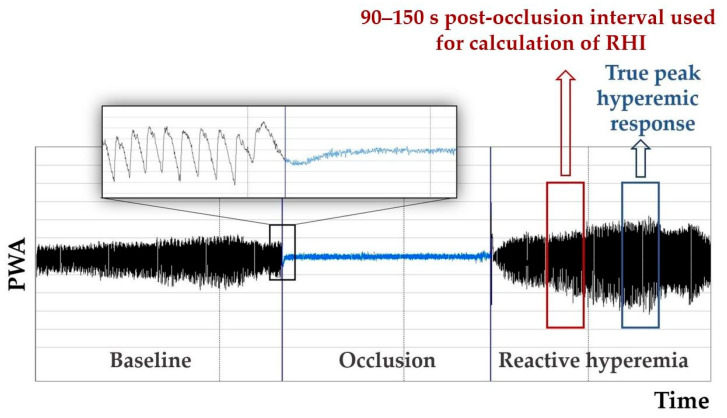
Late onset of peak hyperemic response in healthy adolescent. Due to considerable variability in time to onset of peak hyperemia in the pediatric population, true peak hyperemic response can be missed if evaluated using a standard time period of 90–150 s post occlusion. RHI, reactive hyperemia index; PWA, pulse wave amplitude.

In the pediatric population, considerable variation in the onset of peak hyperemic response was found (Figure 1) [10]; therefore, the application of individually calculated parameters was recommended using the average PWA of the 30 s intervals provided by the PAT software [28]:

*Peak response* is calculated using the 30 s post-occlusion interval with the highest PWA (instead of a fixed 90–150 s period) and corresponding interval from the control arm, and is considered to better reflect the true peak hyperemic response [28]. 

*Time to peak response* is the time between the release of the occlusion and the midpoint of the 30 s peak response interval [28]. 

The *overall hyperemic response* was analyzed using the ratio of the post-occlusion amplitude (from each 30 s interval) to the average baseline PWA and then divided by the same ratio in the contralateral finger, resulting in a PAT ratio for each 30 s interval over the entire 5 min post-occlusion period and controlled for the baseline PWA [10]. The area under the curve (AUC) was calculated using GraphPad Prism 9 and the natural log transformation of AUC was used in statistical analysis. 

### 2.4. Statistical Analysis

The data were analyzed using the statistical software package SYSTAT (Cranes Software International Ltd., San Jose, CA, USA). The normality of data was assessed using the Shapiro–Wilk test. The correlation analyses were performed using Pearson and Spearman correlation.

Data were analyzed using linear regression modeling with Ln AUC as a dependent variable and age, male sex (dummy coded), BMI, mean BP from 24 h ABPM, serum glucose, HDL, LDL, TG, AST, ALT, GGT, ALP, and DHEAS as candidate predictors. The subsets of the variables that best contributed to the prediction of Ln AUC were assessed using Mallows Cp statistics as the best criterion. The presence of multicollinearity was checked using the assessment of the variance inflation factor (VIF). 

Additionally, we tested the indices of reactive hyperemia for the ability to detect the differences in endothelial function between the evaluated groups using one-way analysis of variance ANOVA with Fisher’s least significant difference post-hoc test and Kruskal–Wallis test. Probabilities of *p* < 0.05 were considered significant.

## 3. Results

### 3.1. Between-Groups Comparison of the Indices of Reactive Hyperemia

**Figure 2:**
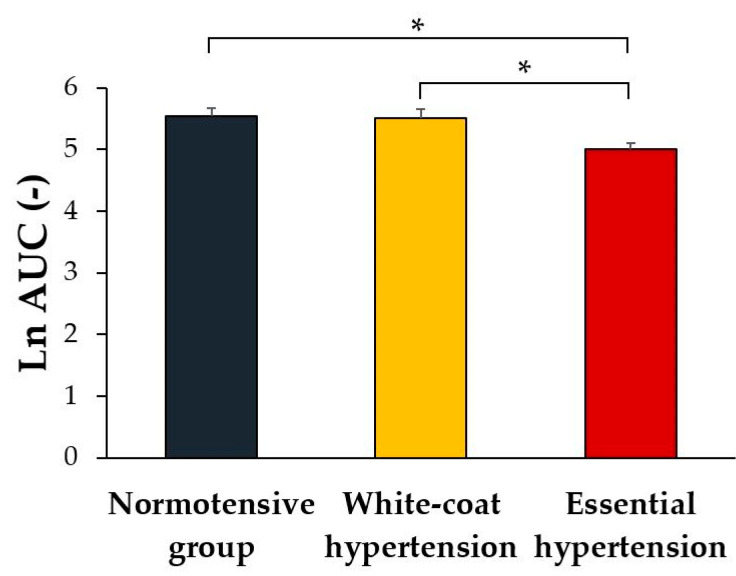
Log-transformed area under the curve (Ln AUC) for the overall hyperemic response in adolescents with normal blood pressure, white-coat hypertension, and essential hypertension. *, *p* < 0.05. Values are expressed as mean ± SEM.

Ln AUC, reflecting the overall hyperemic response, was the only parameter that showed a significant effect of group (normotensives vs. white-coat hypertensives vs. essential hypertensives) on endothelial function (*F*[2] = 3.622, *p* = 0.030). Post-hoc analysis revealed that Ln AUC was significantly lower in the essential hypertension group compared to the normotensive and white-coat hypertension groups (*p* = 0.024, *p* = 0.032, respectively, Figure 2). No significant difference was found between white-coat hypertension and the normotensive group (*p* = 0.903).

Parameters Ln RHI, FRHI, peak response, and time to peak response showed no significant effect of group (H[2] = 0.963, *p* = 0.618; H[2] = 0.979, *p* = 0.613; H[2] = 0.283, *p* = 0.868; H[2] = 1.704, *p* = 0.427, respectively, Table 1).

### 3.2. Associations of the Indices of Reactive Hyperemia with Mean BP

**Figure 3:**
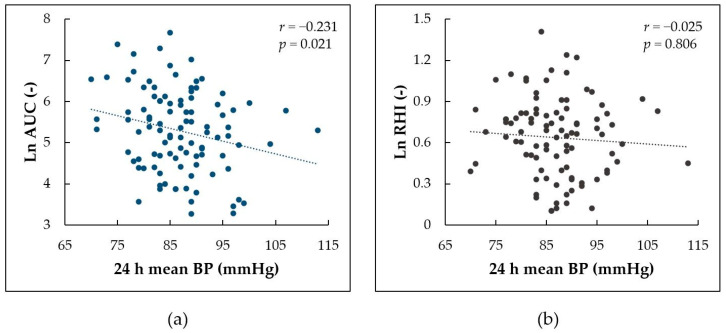
Correlation between mean blood pressure (BP) from 24 h ambulatory blood pressure monitoring and (**a**) log-transformed area under the curve (Ln AUC) for the overall hyperemic response; (**b**) log-transformed reactive hyperemia index (Ln RHI).

First, we evaluated which of the indices of reactive hyperemia best corresponds with mean BP from 24 h ABPM using correlation analyses. Ln AUC, reflecting the overall hyperemic response, was the only parameter significantly correlating with mean BP from ABPM (*r* = −0.231, *p* = 0.021, Figure 3a). Ln RHI, FRHI, peak response, and time to peak response did not show significant correlation with mean BP (*r* = −0.025, *p* = 0.806, Figure 3b; *r* = −0.145, *p* = 0.150; *r* = 0.144, *p* = 0.153; *r* = −0.052, *p* = 0.606, respectively). Therefore, only Ln AUC was further used in regression analysis.

Second, we compared the difference in associations between the overall hyperemic response assessed using Ln AUC and mean BP assessed using two methods—24 h ABPM and standard single oscillometric measurement. While mean BP from 24 h ABPM correlated significantly with Ln AUC (*r* = −0.231, *p* = 0.021), mean BP from a single measurement did not reach statistically significant correlation (*r* = −0.181, *p* = 0.072).

### 3.3. Regression Analysis

The linear regression model with age, sex, mean BP from 24 h ABPM, DHEAS, and AST best predicted overall hyperemic response assessed using Ln AUC (R^2^ = 0.225). The overall hyperemic response evaluated using Ln AUC showed a significant negative association with age of −0.125 year^−1^ (*p* = 0.039), 24 h mean BP of −0.033 mmHg^−1^ (*p* = 0.011), and AST of −1.561 µkat^−1^·L (*p* = 0.033) and a significant positive association with DHEAS of 0.062 µmol^−1^·L (*p* = 0.027). When compared to girls, boys showed a tendency for lower Ln AUC by 0.421; however, this relationship did not reach statistical significance (*p* = 0.102). The regression analysis results are presented in Table 2.

### 3.4. Correlation between BMI and Indices of Reactive Hyperemia

**Figure 4:**
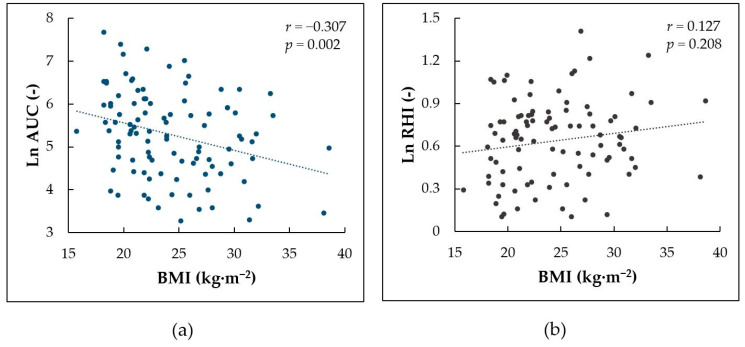
Correlation between body mass index (BMI) and (**a**) log-transformed area under the curve (Ln AUC) for the overall hyperemic response; (**b**) log-transformed reactive hyperemia index (Ln RHI).

BMI showed significant negative correlation with Ln AUC (*r* = −0.307, *p* = 0.002, Figure 4a) but no significant correlation with Ln RHI, FRHI, peak response, or time to peak response (*r* = 0.127, *p* = 0.208, Figure 4b; *r* = −0.047, *p* = 0.643; *r* = −0.092, *p* = 0.360; *r* = −0.111, *p* = 0.272, respectively).

## 4. Discussion

The major finding of this study is that early signs of impaired microvascular endothelial function can be found already in adolescents with newly diagnosed essential hypertension. However, no evidence was found for microvascular alterations in white-coat hypertensives. Secondly, we found that a detailed analysis of the overall magnitude of hyperemic response assessed by PAT could offer a more robust method compared to the standard evaluation of RHI, with the capability to reflect the effects of distinct physiological mechanisms on microvascular endothelium.

### 4.1. Endothelial Function in Pediatric Hypertension

It is well known that hypertension is associated with an increased risk of adverse cardiovascular events in adulthood. Recent studies also emphasized the link between elevated BP and target organ damage in the pediatric population, with abnormal vascular structure and function representing the key mechanisms identifiable already in asymptomatic individuals [29]. In children and adolescents, particular attention should be focused on the risk associated with white-coat hypertension, which is much more common than in the adult population [30]. Regarding the endothelium, macrovascular endothelial dysfunction assessed using FMD was previously found in adolescents with both essential and white-coat hypertension [21]. In the present study, microvascular endothelial function was significantly impaired in adolescents with essential hypertension but, surprisingly, not in those with white-coat hypertension. This discrepancy could be related to certain differences between FMD and PAT methods. Although reactive hyperemia evaluated using PAT was found to be significantly associated with the results from FMD, it seems to be less affected by the activity of NO and, to a greater extent, by the mechanisms involving the effect of adenosine, prostaglandin, endothelium-derived hyperpolarizing factors, potassium, pH, hydrogen peroxide, myogenic response, and microvascular structure [6,31]. Thus, although both methods are closely related, it seems that their outcomes may be considered complementary rather than interchangeable [32]. The clinical relevance of this effect remains to be established. It is possible that each method will show prognostic value specific for distinct spectra of adverse health outcomes associated with endothelial dysfunction. For example, the critical role of microvascular function has been suggested in the progression of cerebral small vessel disease and development of ischemic stroke, particularly in subtypes with early onset in young adults, e.g., cryptogenic ischemic stroke [33,34].

In accordance with the present findings, arterial stiffness evaluated using cardio-ankle vascular index was previously found to be increased in adolescents at the onset of essential hypertension, while those with white-coat hypertension showed only a non-significant intermediate degree of arterial stiffening [35]. It is important to note that endothelial function and arterial stiffness measure different properties of arterial trees and are affected by risk factors in a non-uniform fashion; therefore, a detailed analysis of their interactions could help to understand the pathomechanisms of cardiovascular risk in youth [29].

### 4.2. Measures of Microvascular Endothelial Function—RHI vs. AUC

In this study, analysis of the AUC for the hyperemic response showed better discrimination of the differences in endothelial function between normotensive and hypertensive adolescents and better correlation with out-of-office BP compared to the conventional evaluation of RHI. This finding could be related to the effect of BP hyperreactivity on the parameters of PAT in adolescence, described by Radtke et al. [36]. In their study, normotensive adolescents characterized as BP hyperreactors showed markedly higher RHI, peak response, and a shorter time to peak compared to the control group matched to age, sex, pubertal status, and height. After a short time of physical exercise, these differences were abolished. They speculated that higher RHI at rest could be related to lower baseline PWA reflecting adrenergic activation, which was diminished by post-exercise vasodilation [36]. On the other hand, a shorter time to peak response in BP hyperreactors points to the importance of methodological consideration of a more complex analysis of microvascular function. Traditionally, RHI in the PAT method is calculated using a fixed post-occlusion time interval of 90–150 s after the release of arterial occlusion [9]. In children and adolescents, endothelial function is still under development and the peak hyperemic response was found to be reached between 150 and 204 s; thus, the true peak reactive hyperemia may be missed in this age group when evaluated using RHI [10,28]. Moreover, if the pathophysiological mechanism of the studied risk factor (e.g., BP hyperreactivity) leads to a shorter time to peak, this effect may overlay the negative effect on the overall hyperemic response. In our study, the interquartile range of time to peak response was within 90–240 s. Thus, the interval of true peak reactive hyperemia was missed in a significant part of the participants when evaluated using RHI. This methodological pitfall has been recently considered a principal limitation in the study of pediatric endothelial dysfunction using PAT under pathological conditions [12].

### 4.3. The Effect of BMI and Aspartate Aminotransferase Levels

Regarding the effect of body weight, a previous study showed a positive correlation between BMI and RHI in healthy non-obese children and adolescents [13]. However, this effect could be attributed to a parallel increase in RHI and BMI within pubertal development, which was the only independent predictor of RHI in regression analysis. In our study, while RHI showed no significant correlation with BMI, the AUC of hyperemic response showed a significant negative correlation. This finding could be related to the negative association between AST and microvascular endothelial function indexed by AUC.

AST is an important enzyme in the metabolism of amino acids, energy management, protein synthesis, and other metabolic processes, with the highest activity found in the heart, liver, skeletal muscle, kidney, and brain [37]. Formerly, AST was used as a biochemical marker in the diagnosis of myocardial infarction. Today this application is obsolete; however, the relationship between AST and cardiovascular disease remains at the center of interest. Regarding endothelial dysfunction, the most common mechanism of the association with AST levels seems to be mediated by the presence of non-alcoholic fatty liver disease, resulting in increased synthesis of proinflammatory and procoagulant factors and increased oxidative stress [37,38]. Hepatic steatosis is common in children with obesity [39]; thus, we can assume that this mechanism could play a significant role in endothelial dysfunction in overweight hypertensives in our study.

### 4.4. The Effect of Age, Pubertal Development, and Out-of-Office BP

The blood concentration of DHEAS reflecting pubertal advancement showed a positive effect on endothelial function evaluated using AUC, which is in accordance with other studies [13,15]. Interestingly, the effect of age itself, when evaluated together with pubertal development, showed a negative association with endothelial function. This finding points to a dominant role of pubertal hormones in the maturation of the microvascular endothelium.

In this study, AUC was the only parameter of microvascular endothelial function that showed a significant negative correlation with mean BP from 24 h BP measurements. Moreover, 24 h ABPM showed a much stronger association with AUC compared to BP taken in the clinical settings at the time of examination, which did not reach statistical significance. This is an important methodological point in the context of the white-coat effect, which is relatively frequent in the pediatric population [30].

A summary of the recent findings on the effects of adiposity, age, and BP on the parameters of PAT is presented in Table 3.

## 5. Conclusions

To our best knowledge, this is the first study reporting early signs of microvascular endothelial dysfunction evaluated using PAT in adolescents with newly diagnosed essential hypertension. In white-coat hypertensives, no significant alterations were found, which is in contrast to previously found macrovascular endothelial dysfunction evaluated using FMD. This discrepancy could be related to distinct physiological mechanisms involved in reactive hyperemia of microvascular and macrovascular circulation. The clinical relevance of this difference remains to be established. Detailed analysis of hyperemic response using evaluation of the overall magnitude indexed by AUC provided a more robust method compared to the conventional evaluation of RHI. This method could help to exclude the effect of interindividual differences in the time necessary to reach peak hyperemic response, which seems to represent the principal limitation in the study of pediatric endothelial function using PAT.

## Figures and Tables

**Table 1:** Parameters of hyperemic response in normotensive subjects, adolescents with white-coat and essential hypertension.

	Normotensive Group (*n* = 27)	White-Coat Hypertension Group (*n* = 28)	Essential Hypertension Group (*n* = 45)
Ln RHI (-)	0.690 (0.450–0.810)	0.630 (0.238–0.943)	0.680 (0.510–0.825)
FRHI (-)	0.676 (0.502–0.932)	0.711 (0.183–0.955)	0.634 (0.367–0.818)
Peak response (-)	2.280 (1.792–2.565)	2.303 (1.453–2.780)	2.199 (1.749–2.580)
Time to peak response (s)	150.0 (90.0–240.0)	180.0 (120.0–232.5)	120.0 (90.0–210.0)

Ln RHI, log-transformed reactive hyperemia index; FRHI, variation of reactive hyperemia index used in Framingham Heart Studies. Values are expressed as median (IQR).

**Table 2:** Estimated effects of age, sex, BP, DHEAS, and AST on overall hyperemic response assessed using Ln AUC.

Parameter	Coefficient	Std. Error	Units	*p*-Value
Intercept	10.786	1.474	-	<0.001
Age	−0.125	0.060	year^−1^	0.039
Male sex	−0.421	0.254	-	0.102
Mean BP (24 h ABPM)	−0.033	0.013	mmHg^−1^	0.011
DHEAS	0.062	0.028	µmol^−1^·L	0.027
AST	−1.561	0.719	µkat^−1^·L	0.033

Ln AUC, the log-transformed area under the curve of the overall hyperemic response; BP, blood pressure; ABPM, ambulatory blood pressure monitoring; DHEAS, dehydroepiandrosterone sulphate; AST, aspartate aminotransferase. Male sex is dummy-coded and indicates the difference between males and females.

**Table 3:** Studies on the effects of adiposity, age, and blood pressure on microvascular endothelial function assessed using PAT in children and adolescents.

Studied Population/Evaluated Mechanism	Findings	Reference
540 children aged 7–17 years (80 with overweight, 73 with obesity), the effect of the obesity and cardiometabolic risk indicators	Increase of RHI, F-RHI, and Peak response with age Decrease of RHI, F-RHI, and Peak response with diastolic BP Decrease of RHI with TG in obese individuals	He et al., 2022 [40]
27 overweight/obese and 25 normal-weight adolescents aged 12–20 years	Decrease of RHI with diastolic BP Lower RHI in overweight/obese subjects	Pareyn et al., 2015 [41]
20 children aged 9–19 years with systemic lupus erythematosus, the effect of nocturnal BP dipping	Lower RHI in patients with nocturnal BP non-dipping pattern assessed using 24 h ABPM	Chang et al., 2020 [42]
62 obese and 61 normal weight children aged 8–18 years	Increase of RHI with age in lean but not obese children	Tryggestad et al., 2012 [43]
36 obese adolescents aged 11–19 years, the effect of the presence of NAFLD	Decrease of RHI with hepatic fat content	Bacha et al., 2017 [44]
29 obese and 29 non-obese adolescents and young adults aged 12–23 years, the effect of hemodynamic parameters	Positive correlation between RHI and systemic vascular resistance, no significant difference in RHI between obese and non-obese subjects	Czippelova et al., 2019 [45]
130 obese children aged 8–18 years, the effect of weight loss	Increase of RHI and decrease of Time to peak response after weight loss	Jacobs et al., 2021 [46]

ABPM, ambulatory blood pressure monitoring; BP, blood pressure; F-RHI, Framingham reactive hyperemia index; NAFLD, nonalcoholic fatty liver disease; PAT, peripheral arterial tonometry; RHI, reactive hyperemia index; TG, triglycerides.

## Data Availability

Data will be available on request to the corresponding author.
